# Clinically Relevant Mutant DNA Gyrase Alters Supercoiling, Changes the Transcriptome, and Confers Multidrug Resistance

**DOI:** 10.1128/mBio.00273-13

**Published:** 2013-07-23

**Authors:** Mark A. Webber, Vito Ricci, Rebekah Whitehead, Meha Patel, Maria Fookes, Alasdair Ivens, Laura J. V. Piddock

**Affiliations:** Antimicrobial Agents Research Group, School of Immunity and Infection, Institute of Microbiology and Infection, The University of Birmingham, Edgbaston, Birmingham, United Kingdom^a^; The Wellcome Trust Sanger Institute, Wellcome Trust Genome Campus, Hinxton, Cambridge, Cambridgeshire, United Kingdom^b^; Centre for Immunity, Infection and Evolution, University of Edinburgh, Edinburgh, United Kingdom^c^

## Abstract

Bacterial DNA is maintained in a supercoiled state controlled by the action of topoisomerases. Alterations in supercoiling affect fundamental cellular processes, including transcription. Here, we show that substitution at position 87 of GyrA of *Salmonella* influences sensitivity to antibiotics, including nonquinolone drugs, alters global supercoiling, and results in an altered transcriptome with increased expression of stress response pathways. Decreased susceptibility to multiple antibiotics seen with a GyrA Asp87Gly mutant was not a result of increased efflux activity or reduced reactive-oxygen production. These data show that a frequently observed and clinically relevant substitution within GyrA results in altered expression of numerous genes, including those important in bacterial survival of stress, suggesting that GyrA mutants may have a selective advantage under specific conditions. Our findings help contextualize the high rate of quinolone resistance in pathogenic strains of bacteria and may partly explain why such mutant strains are evolutionarily successful.

## IMPORTANCE

Fluoroquinolones are a powerful group of antibiotics that target bacterial enzymes involved in helping bacteria maintain the conformation of their chromosome. Mutations in the target enzymes allow bacteria to become resistant to these antibiotics, and fluoroquinolone resistance is common. We show here that these mutations also provide protection against a broad range of other antimicrobials by triggering a defensive stress response in the cell. This work suggests that fluoroquinolone resistance mutations may be beneficial under a range of conditions.

## Introduction

Bacterial chromosomal DNA exists in a complicated, condensed state in which the nucleoid consists of a large number of domains of independently supercoiled DNA (1–3). Supercoiling of chromosomal DNA is not fixed, and the integration of supercoiling changes as a messenger of environmental stress in concert with other regulatory networks and consequent transcriptome alterations is important (4, 5). The level of supercoiling of DNA in *Escherichia coli* and *Salmonella enterica* is determined by the opposing actions of DNA gyrase and topoisomerase I (6). DNA gyrase is a type II topoisomerase which introduces negative supercoils into DNA in an ATP-dependent manner and exists as a heterotetramer of two GyrA and two GyrB monomers (7). In contrast, topoisomerase I acts to relax supercoiled DNA (8). Chromosomal supercoiling affects several crucial cellular processes, including transcription, replication, and recombination; thus, alterations in the degree of global supercoiling can have numerous phenotypic implications (9). For example, Peter et al. (10) demonstrated that approximately 7% (over 300 genes) of the *E. coli* transcriptome was sensitive to alterations in supercoiling and that genes induced upon chromosomal relaxation were dispersed around the chromosome. These were associated with up- and downstream areas of low AT content. Similarly, *Streptococcus pneumoniae* has been shown to alter global transcription in response to gyrase inhibition (11), and it has also been shown that the supercoiling-responsive genes reside in 15 large physical clusters of genes which are flanked by regions rich in AT content. A previous proteomic study (12) had also shown wide-scale changes to protein abundance in response to mutation of *gyrB* and *topA*. Long-term evolution experiments with *E. coli* have implicated genes which control supercoiling as being subject to selection, with mutations in *topA* and *fis* occurring in multiple lineages and a consequent increase in supercoiling levels being seen (13). This has been suggested to be due to an increase in evolutionary flexibility associated with these mutations rather than any direct fitness benefit (14).

Gyrase is an essential enzyme required for viability of bacterial cells and has proved an attractive target for various antibiotics, including the quinolones. Quinolone-mediated cell killing is complicated and involves formation of a drug-gyrase-DNA complex, which ultimately results in the release of double-stranded DNA and, as a result, fragmentation of the chromosome and cell death (15, 16). Two pathways by which the DNA breakage occurs have been described, one which requires active protein synthesis (the chloramphenicol-sensitive pathway, named due to the ability of chloramphenicol to inhibit killing) and one which does not (the chloramphenicol-insensitive pathway) (17, 18). Older, nonfluorinated quinolones act via the first pathway, and newer fluoroquinolones can act via the second pathway. Recently, the endogenous generation of reactive oxygen species in response to treatment with bactericidal antibiotics has been proposed to aid killing of *E. coli* by antibiotics (19, 20). This has been validated for quinolones, although this effect is restricted to the chloramphenicol-sensitive pathway of cell death, and recently, the impact of reactive oxygen in cell killing has been disputed (18, 21, 22).

In Gram-negative bacteria, resistance to quinolone antibiotics arises from mutations within *gyrA* and/or *gyrB* and/or *parC* and *parE*. The latter genes encode components of the topoisomerase IV enzyme, which is homologous to gyrase and a secondary target for quinolones in Gram-negative bacteria. Mutations are commonly clustered within small regions of these genes, the quinolone resistance-determining regions (23–26). Changes result in structural changes at the target site of the enzyme tetramer which lead to reduced affinity for quinolone drugs and consequent resistance to these agents (27). Substitutions within GyrA are most frequently seen at position 83 or 87, with highly quinolone-resistant strains often also possessing additional substitutions within GyrB and/or ParC as well as non-quinolone-specific mechanisms of resistance, such as increased expression of multidrug efflux pumps (28). These mutations can individually confer a fitness cost *in vitro*, although often in combination, and there are compensation and amelioration of the initial fitness impact of each mutation; quinolone-resistant mutants are also stable and often able to survive well in *in vivo* competition assays (29, 30). Previously, we showed that selection of mutants resistant to the biocide triclosan occurred from a *gyrA* mutant background (carrying an Asp87Gly substitution) at a significantly higher frequency than from wild-type strains (31, 32) suggesting that a *gyrA* mutation provides some benefit against triclosan and possibly other bactericidal drugs.

In this study, we explored the effect of specific substitutions at positions 83 and 87 of GyrA on DNA supercoiling, the transcriptome, and the susceptibility of *Salmonella* to bactericidal antibiotics. A single mutation introduced by site-directed mutagenesis to create a substitution within GyrA at position 87 provided protection against some antibiotics. The mutants had altered supercoiling activity, as measured by a plasmid reporter and an altered transcriptome. Among genes differentially expressed in response to *gyrA* mutation were those relevant to antibiotic resistance, indicating that changes to supercoiling are stressful and confer a response which is protective against bactericidal antibiotics.

## RESULTS

### Effects of mutation in topoisomerase genes upon antibiotic susceptibility.

Site-directed mutagenesis of *gyrA* resulted in a substitution of serine with phenylalanine at position 83 of GyrA in L821 and a substitution of glycine for aspartate at codon 87 in L825. L821 and L825 demonstrated identical patterns of susceptibility to antimicrobial agents (Table 1). Both mutants were significantly less susceptible to ciprofloxacin (MIC = 0.5 µg/ml), nalidixic acid (MIC > 256/ml), and triclosan (MIC = 0.25 µg/ml) than their parent, SL1344.

**TABLE 1:** Strains used in this study, antimicrobial susceptibility, topoisomerase genotype and relative fitness^*a*^

Strain	GyrA substitution	Relative fitness^*b*^	MIC (µg/ml)								
			Cip	Nal	Chl	Tet	Kan	Gent	Amp	Tric	EtBr
SL1344	None (wild type)	1.00 (0.02)	0.03	4	2	1	2	0.5	0.5	0.06	>512
L821	Ser83Phe	0.96 (0.04)	0.5	>256	4	1	2	0.5	1	0.25	>512
L825	Asp87Gly	0.92 (0.03)	0.5	>256	4	1	2	0.5	1	0.25	>512

^a^ Cip, ciprofloxacin; Nal, nalidixic acid; Chl, chloramphenicol; Tet, tetracycline; Kan, kanamycin; Gent, gentamicin; Amp, ampicillin; Tric, triclosan; EtBr, ethidium bromide.

^b^ Defined as 1 + S (the selection coefficient). Numbers in parentheses are standard deviations.

**FIG 1:**
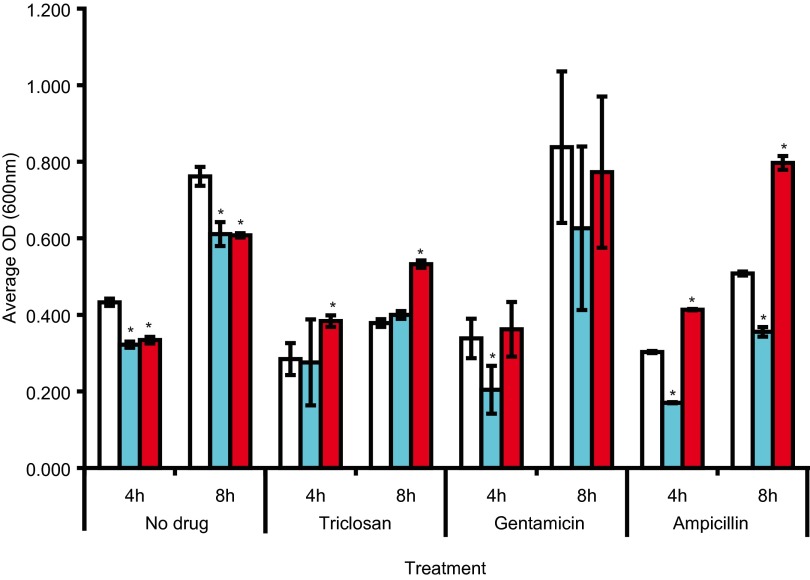
Differences in growth between strains and the impact of the addition of half the MIC for SL1344 of ampicillin, gentamicin, and triclosan on growth. The graph shows average culture OD values at 4 and 8 h postinoculation; averages that are statistically significantly different from those of the parent strain, SL1344, are marked by asterisks. Open bars indicate SL1344, blue bars indicate L821 (GyrA Ser83Phe), and red bars indicate L825 (GyrA Asp87Gly).

**FIG 2:**
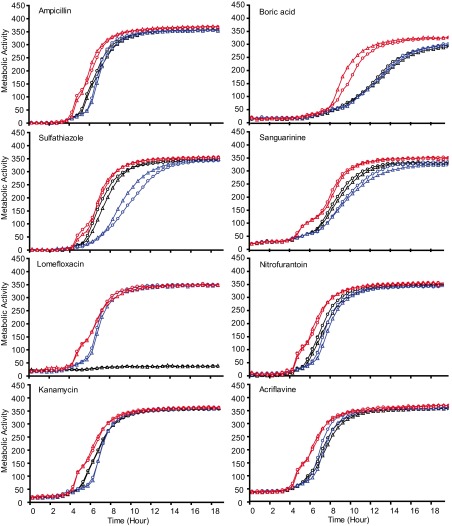
Respiratory activity of SL1344 (black lines), L821 (blue lines), and L825 (red lines) in the presence of eight separate antimicrobials in the Biolog Phenomicroarray. Circles and triangles represent separate biological replicates in each experiment.

Compared to SL1344 and in the absence of antibiotics, growth kinetics showed that both mutants grew more slowly than SL1344 (Fig. 1; also, see Fig. S1 in the supplemental material). Growth kinetics were also determined in the presence of subinhibitory concentrations (0.5× the MIC) of antibiotics. L825 (GyrA Asp87Gly) grew better than SL1344 and L821 (GyrA Ser83Phe) when exposed to ampicillin and triclosan but not when exposed to gentamicin (Fig. 1). A similar pattern was also observed at both 30 and 42°C. To further determine whether L825 is less susceptible to antimicrobials, we used the Phenomicroarray (Biolog) to measure respiration (growth) rates of each strain in the presence of over 200 antimicrobials. These data showed that both L821 and L825 were able to respire better then SL1344 in the presence of a range of quinolones. In addition, L825 showed greater respiratory activity in the presence of 18 other agents, including beta-lactams, folate synthesis inhibitors, aminoglycosides, and toxic anions (Table 2). L821 did not, however, show any increase in respiratory activity in the presence of the agents tested compared to SL1344. Figure 2 shows a selection of agents of the different classes where differential respiratory activity between the strains was observed. L825 did not show decreased respiratory activity compared to SL1344 with any of the agents tested. To examine whether the increased growth/respiration of L825 seen in the presence of ampicillin was a homogenous property of the population, we used flow cytometry to investigate the cellular viability of populations. Compared with SL1344 and L821, a higher percentage of cells of L825 remained viable and maintained a membrane potential after a 4-h exposure to ampicillin. When exposed to 25 µg/ml of ampicillin (50× the MIC) for 4 h, approximately 45% of the SL1344 population was dead, whereas <25% of the population of L825 was dead (see Fig. S2 in the supplemental material). L821 did not show a significant change in susceptibility to killing by ampicillin relative to SL1344 or L825. These data indicate that the nature of *gyrA* mutation is important in surviving exposure to antibiotics.

**TABLE 2:** Toxic compounds to which mutation of Asp87 to Gly of gyrase provided a survival benefit

Group and compound	Mechanism of action
Beta-lactams	
Cefoxitin	Cell wall synthesis
Ampicillin	Cell wall synthesis
Carbenicillin	Cell wall synthesis
	
Folate synthesis inhibitors/antagonists	
2,4-Diamino-6,7-diisopropylpteridine	Folate antagonist
Sulfamethazine	Folate synthesis inhibitor
Sulfadiazine	Folate synthesis inhibitor
Sulfathiazole	Folate synthesis inhibitor
Sulfamethoxazole	Folate synthesis inhibitor
	
Quinolones	
Lomefloxacin	DNA gyrase inhibitor
Enoxacin	DNA gyrase inhibitor
Ofloxacin	DNA gyrase inhibitor
Nalidixic acid	DNA gyrase inhibitor
Oxolinic acid	DNA gyrase inhibitor
	
Protein synthesis inhibitors	
Neomycin	Inhibitor of 30S ribosomal subunit
Kanamycin	Inhibitor of 30S ribosomal subunit
	
Toxic anions	
Boric acid	Toxic anion
Sodium cyanate	Toxic anion
Sodium arsenate	Toxic anion, phosphate analog
Sodium metavanadate	Toxic anion, phosphate analog
Sodium orthovanadate	Toxic anion, phosphate analog
	
Other	
Rifampin	RNA polymerase inhibitor
Sanguinarine	Membrane potential
Nitrofurantoin	Reactive attack of macromolecules

### Impact of gyrase mutations on *in vitro* bacterial fitness.

Growth competition assays of each of the two *gyrA* mutants against SL1344 were used to determine the relative fitness of L821 (GyrA Ser83Phe) and L825 (GyrA Asp87Gly). Both mutations imposed a small cost; the competitive fitness of L825 was slightly worse than that of L821, with a relative fitness compared to SL1344 of 0.92 and 0.96, respectively (Table 1).

### Cellular permeability and efflux.

To determine whether the increased survival of L825 (GyrA Asp87Gly) in the presence of antibiotics compared to L821 (GyrA Ser83Phe) and SL1344 was due to reduced cellular permeability and/or increased efflux activity, the accumulation of the intercalating fluorescent dye Hoechst 33342 by all strains was measured (see Fig. S3 in the supplemental material). Hoechst 33342 is a substrate for the major multidrug resistance system of *Salmonella*, AcrAB-TolC mutants overproducing this system accumulate significantly less dye than wild-type strains (33). Accumulation of Hoechst was significantly higher in L825 than SL1344 and L821. These data indicate that the decreased susceptibility to antibiotics of L825 is not a result of a decrease in cell envelope permeability or increased efflux activity.

### Alterations to supercoiling in L821 and L825.

**FIG 3:**
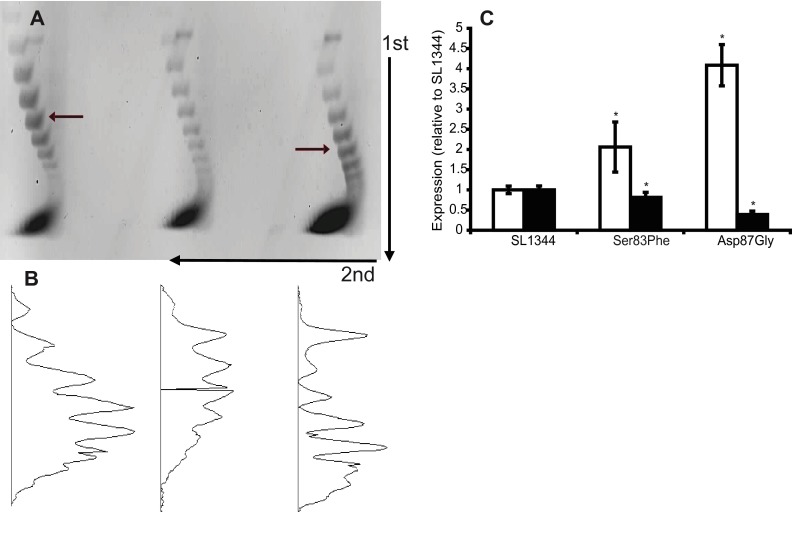
Supercoiling activity and expression of *gyrA* and *topA* by SL1344, L821, and L825. (A) Separation of pBR322 isolated from each strain on a representative 0.9% agarose gel containing 25 mg/liter chloroquine. Red arrows indicate altered supercoiling; black arrows indicate direction of electrophoresis. (B) Densitometry plots of the lanes in the gel from panel A. (C) Expression of *gyrA* and *topA*, measured by comparative RT-PCR. Error bars indicate standard deviations, and asterisks indicate values that are significantly different from the value for SL1344.

The level of supercoiling of plasmid pBR322 within SL1344, L821 (GyrA Ser83Phe), and L825 (GyrA Asp87Gly) was determined and found to differ in the two mutants (Fig. 3). Compared to SL1344, both mutants showed a shift in the topoisomer band patterns, indicating a reduction in the degree of negative supercoiling of DNA. This reduction was more marked for L825 (13% reduction in activity) than for L821 (5% reduction in activity) (Fig. 3A and B). These results indicate that mutation within *gyrA* reduces gyrase activity and as a result lessens the degree of supercoiling of DNA within the cell (as found previously with *E. coli* [34]). Comparative reverse transcription-PCR (RT-PCR) (Fig. 3C) of *topA* and *gyrA* showed a significant increase in *gyrA* expression in both L821 (~2-fold) and L825 (~4-fold) and significant decreases in *topA* expression in both mutants (~1.25- and 2.5-fold, respectively) compared to SL1344. These data are consistent with a response to altered supercoiling (34). The larger shift in the ratio of *gyrA* to *topA* expression seen in L825 than in L821 suggests a greater functional consequence of the Asp87Gly substitution in this mutant (Fig. 3).

### Transcriptomic changes in L825.

As efflux and cellular permeability did not appear to be involved in the phenotype of L825 (decreased susceptibility to a range of antimicrobials), the transcriptome of L825 (GyrA Asp87Gly) was determined and compared to that of SL1344 in order to identify candidate genes involved in enhanced antibiotic survival. Expression of 499 genes was statistically significantly altered in L825 compared to SL1344. Among those genes with altered expression were a number previously implicated in resistance to antibiotics or general stress (Table 3; also, see Tables S1 and S2 in the supplemental material).

**TABLE 3:** Genes involved in stress responses with significantly altered expression in L825

Function	Gene	Common name	Annotation	Expression (fold change relative to SL1344)
Transcription control	SL2603	*rseA*	Sigma E factor negative regulatory protein	1.9
	SL2604	*rpoE*	RNA polymerase sigma-E factor (sigma-24)	2.0
	SL2903	*rpoS*	RNA polymerase sigma subunit RpoS (sigma-38)	2.7
	SL3185	*rpoD*	RNA polymerase sigma-70 factor	0.7
	SL3292	*rpoN*	RNA polymerase sigma-54 factor (sigma-N)	1.9
	SL3707	*rpoZ*	DNA-directed RNA polymerase omega chain	0.5
	
Multidrug efflux	SL2798	*emrR*	Putative transcriptional regulator	6.6
	SL2798	*emrA*	Multidrug efflux system	6.0
	SL0469	*acrA*	Acriflavine resistance protein A precursor	0.009
	
Stress response	SL2809	*recA*	RecA protein	2.9
	SL0012	*dnaK*	DnaK protein (heat shock protein 70)	0.09
	SL0187	*dksA*	DnaK suppressor protein	0.5
	
DNA condensation	SL4109	*hupA*	Histone-like DNA-binding protein HU-alpha	0.5
	SL0445	*hupB*	Histone-like DNA-binding protein HU-beta	0.3

Expression of housekeeping sigma factor, *rpoD* was reduced twofold in L825, whereas the expression of stress-responsive sigma factors *rpoS*, *rpoE*, and *rpoN* were all significantly increased (2- to 3-fold) (Table 3). This expression pattern supports the hypothesis that altered supercoiling acts as a signal of stress and that specific sigma factors are induced in response to alterations in supercoiling. The expression of *recA* was also increased 2.9-fold in L825, indicating that a DNA damage stress response has been induced in L825.

Altered expression of genes encoding components of multidrug efflux pumps was seen in L825 (Table 3). The expression of *acrA* was significantly down-regulated (>100-fold); these data agree with the increased accumulation of efflux substrate Hoechst 33342 seen in L825 relative to L821 and SL1344 (see Fig. S3 in the supplemental material) and also correlated well with data from an *acrAB-gfp* reporter experiment. The decreased expression of efflux genes may be explained in part by the decreased level of *rpoD* in L825, as RpoD has been suggested to control *acrAB* expression in *E. coli* (35). Increased expression (>6-fold) of *emrR* and *emrA* was also seen in L825; however, inactivation of *emrAB* (by insertional activation with a chloramphenicol resistance cassette) in L825 and L821 resulted in no difference in the MIC of the compounds listed in Table 1, again suggesting efflux is not relevant to the decreased antimicrobial susceptibility of L825.

It has been suggested that there is a link between susceptibility to bactericidal antibiotics (including quinolones) and central metabolism, with aerobic metabolism pathways involved in production of reactive oxygen species, which are involved in cell killing (20, 36). Expression of genes involved in energy generation in L825 was also different from that in SL1344 (see Tables  S1 and S2 in the supplemental material). These included genes encoding NADH dehydrogenase (*nuoHIJKLM*, repressed three- to fourfold) and ATP synthase components (*atpABCDEFG*, repressed twofold) indicating a reduction in activity of the electron transport chain. There was no compensatory increase in genes involved in anaerobic metabolism. The expression of *arcB*, previously shown to increase in expression in response to reduced activity of the electron transfer chain in anaerobic growth (37), and *orgA*, known to be repressed by oxygen during logarithmic growth (38), was significantly increased in L825 (two- and fourfold, respectively) (see Table S1). These data suggest a switch away from aerobic respiration in L825. In addition to the transcriptomic changes already described, genes encoding structural ribosome components were also substantially and markedly decreased in expression (18 genes repressed two- to six-fold) in L825. Expression of *Salmonella* pathogenicity island II (SPI-II) genes was also markedly reduced, with almost all genes within the island being affected (see Table S2), suggesting that the expression of genes within these regions is sensitive to supercoiling.

In order to identify functionally interactive gene networks the list of genes with significantly altered expression in L825 was analyzed with Visant. This revealed eight networks with gene products predicted to interact. These included networks involved in aerobic metabolism and protein synthesis and secretion, a large group of related transcriptional regulators with varied repression or derepression, and genes involved in molybdenum cofactor biosynthesis. The repression of genes in groups with predicted functional relationships strengthens the transcriptomic observations and indicate that aerobic metabolism and protein synthesis are both influenced by global supercoiling (as indicated by plasmid reporter assays) and that this may be a programmed and coordinated response for these genes. All the gene networks identified as being responsive to gyrase mutation showed repression of most or all members of the network.

### General stress can recapitulate the beneficial effect of the Asp87Gly substitution.

To explore whether the beneficial effect of the Asp87Gly substitution of GyrA may be due to altered supercoiling and artificially indicating the presence of external stressors, flow cytometry was used to determine the survival of populations of SL1344 exposed to ampicillin after heat shock (growth at 42°C) and ciprofloxacin exposure (exposure to one-half the MIC). Figure S4 in the supplemental material shows that both exposures significantly increased the proportion of surviving cells after both 30 min and 4 h. Figure S4 also demonstrates that the relative increase in survival of SL344 to ampicillin treatment after heat shock is significantly greater than that seen with L825, suggesting that L825 demonstrates a constitutive increase in stress response pathways, limiting the capacity for further induction. Data similar to those from the flow cytometry were obtained in conventional colony counting cell viability experiments (see Fig. S4C and D in the supplemental material).

### Measurement of reactive oxygen production in response to antibiotics.

To determine whether a reduced capacity to generate reactive oxygen species was present in L825 (GyrA Asp87Gly) and therefore relevant to its phenotype, the production of reactive oxygen in response to exposure to ampicillin and ciprofloxacin was measured in all three strains (see Fig. S5 in the supplemental material). The results showed minimal change in production of reactive oxygen between the three strains after the addition of ampicillin, although levels were slightly higher in L825 than in the other two strains. Exposure to ciprofloxacin resulted in a lower rate of reactive oxygen production in the two *gyrA* mutants. This observation was expected, as the specific target site mutations they carry lessen the ability of ciprofloxacin to bind to GyrA and hence reduce its antimicrobial action.

### Chromosomal order of differentially expressed genes.

**FIG 4:**
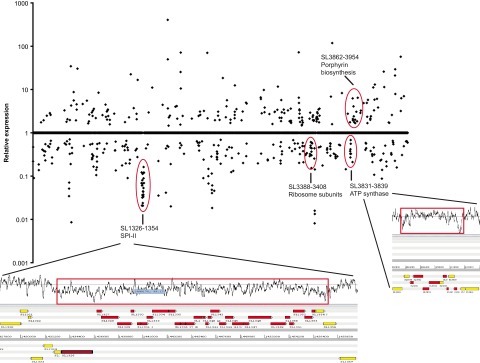
Expression of genes significantly changed with chromosomal order, with genes organized from start to finish of the SL1344 chromosome, left to right. Expression of “1” indicates no significant change; higher values show induced genes, and lower values show repressed genes. Clusters of at least ten consecutive genes with expression altered in a similar manner belonging to at least 2 separate operons are indicated by red circles. Pop-out boxes show the chromosomal context and GC content of two clusters of genes responsive to *gyrA* mutation, the extent of the responsive gene cluster is marked by the boundaries of the red boxes, genes with significantly altered expression are red (repressed) or yellow (derepressed).

To determine whether genes responsive to *gyrA* mutation were organized in physical clusters (as has been reported for streptococci), the chromosomal order of differentially expressed genes was analyzed. While genes with altered expression are distributed across the chromosome, there are clusters of genes which follow common expression patterns (Fig. 4). Clusters were found which correlated to the groups and networks of genes previously identified as demonstrating changed expression. These included repression of groups of genes encoding ribosome subunits, ATP synthase, and SPI-II components (Fig. 4). Analysis of the chromosomal GC content at the borders of these clusters that are responsive to *gyrA* mutation showed they were flanked by short areas of AT-rich DNA, a pattern similar to that observed for supercoiling-responsive clusters in *S. pneumoniae* (39).

## DISCUSSION

We demonstrate here that a common and clinically relevant fluoroquinolone resistance mutation of *gyrA* resulting in an Asp87Gly substitution reduces negative supercoiling and results in large-scale changes to gene expression as well as influencing relative fitness and susceptibility to antibiotics. Mutations within *gyrA* are common in quinolone-resistant pathogenic isolates, and mutants with changes in *topA* or *fis* have been found to increase fitness in *E. coli* in prolonged evolution experiments (13, 14); additionally, *gyrA* mutations in *Campylobacter* have been shown to confer a fitness benefit, and this has been correlated with changes to supercoiling (5). However, it is accepted that mutation within *gyrA* generally results in a small fitness cost in *Salmonella* and *E. coli* (although the two species maintain supercoiling at different levels [4]); therefore, it is unlikely that any direct fitness benefit of *gyrA* mutation contributes to the prevalence of these mutations in these species (40). Here, we compared the impact of two common mutations associated with quinolone resistance and found that a substitution at codon 87 of GyrA (Asp to Gly, to give L825) resulted in a significant phenotype, whereas a substitution at codon 83 (Ser to Phe, to give L821) did not have any impact on supercoiling or tolerance to nonquinolone drugs. Previous work by Barnard and Maxwell analyzed the impact of substitution at these positions in *E. coli* GyrA and also found changes at codon 87 to have a greater impact on supercoiling activity than changes at codon 83 of the enzyme (41). We also investigated whether altered supercoiling in L825 (Asp87Gly) resulted in changes to the transcriptome; mutation of *gyrA* (Asp87Gly) affected approximately 10% of the transcriptome, similar to the 7% of *E. coli* genes found to respond to supercoiling (10). While nearly 500 genes had differential expression in L825, there were clusters of genes which showed altered expression in a consistent manner. These included groups of genes whose products functionally interact and were colocalized on the chromosome. As these clusters were flanked by short AT-rich regions of DNA (Fig. 4), it is conceivable that they are responsive to supercoiling, as has been observed in *E. coli* (10) and *S. pneumoniae* (11). Genes identified as being responsive to *gyrA* mutation included those required for aerobic metabolism, ATP and protein synthesis, and pathogenicity (SPI-II), which showed marked repression, and genes involved in a global response to stress (RpoE, RpoS, and RecA), which were derepressed in L825. The finding in this study that a global stress response (RpoS) is initiated as part of a core change resulting from *gyrA* suggests that this core change may provide a low level of decreased susceptibility to a range of drugs. When overexpressed, RpoS has been shown to contribute to multidrug resistance in *E. coli* (42); in this study, we observed increased expression of *rpoS* in L825. Recently, a relationship between aerobic metabolism, production of endogenous reactive oxygen species, and killing of bacteria by bactericidal drugs was suggested (19, 20, 43). Furthermore, the exposure to a low level of bactericidal antibiotics increased mutation rates due to reactive oxygen production and therefore promoted the emergence of antibiotic resistant mutants. Kohanski et al. (36) demonstrated that exposure of *E. coli* to ampicillin selected mutants that are cross-resistant to other antibiotics, with the highest frequency being seen for norfloxacin resistant mutants. These norfloxacin-resistant mutants were found to carry mutations within *gyrA*. While the work with *E. coli* by Kohanski et al. (36) suggested that exposure to bactericidal antibiotics may increase generic mutation rates and thereby select resistant mutants at an elevated frequency, we and two other teams more recently published data which do not support the reactive-oxygen killing model (21, 22, 44). We show here that the *gyrA* mutation itself influenced susceptibility to nonquinolone antibiotics without further mutation, and we propose that this increased expression of global stress responses rather than decreasing the potential for generation of reactive oxygen. This hypothesis is supported by flow cytometry and viable-count data, which showed that a greater proportion of a population of L825 was able to survive treatment with ampicillin than SL1344—this represents physiological adaptation rather than mutation. In support of our hypothesis, we have previously shown that selection of mutants resistant to triclosan (which targets FabI, not gyrase) occurs at a significantly higher frequency from *gyrA* mutants than wild-type strains (31, 32) indicating that *gyrA* mutation can influence the development of resistance to other antimicrobials.

Our data show that changes to gyrase can alter broad antimicrobial susceptibility as well as the previously described roles in fitness and evolutionary adaptability. This may explain in part the high frequency of quinolone-resistant mutants in nature—these mutants may have a selective advantage in the presence of low levels of environmental antimicrobials, possibly including host-produced antimicrobial molecules. In conclusion, we show that transcription of a large part of the *Salmonella* genome is responsive to altered supercoiling and that supercoiling-sensitive clusters or islands of genes exist in *Salmonella*. Furthermore, our data suggest that supercoiling changes result in multiple gene expression changes, including changes within pathways that contribute to antibiotic resistance, fitness, and pathogenicity of *Salmonella*.

## MATERIALS AND METHODS

### Bacterial strains and plasmids.

Bacteria were routinely grown in Luria-Bertani (LB) broth and on LB agar plates when required and incubated at 37°C. Chemicals were from Sigma-Aldrich (Gillingham, United Kingdom) unless stated otherwise.

### Mutant construction.

L821 and L825 were both created from wild-type strain SL1344 using a suicide vector site-directed mutagenesis method as described by Turner et al. (45). Briefly, mutant *gyrA* alleles from quinolone-resistant mutants of *Salmonella enterica* serovar Typhimurium were amplified and cloned into the suicide vector pJCB12 using appropriate restriction sites before propagation in *E. coli* strain CC118λ*pir*. Plasmid DNA was harvested and introduced into SL1344 by electroporation, and integration of the plasmid was selected for by growth on chloramphenicol-containing agar (25 µg/ml). Ten colonies for each allele were then cultured in LB broth (10 ml) overnight without selection before being plated onto LB agar (without salt) containing 5% sucrose to select for excision and loss of pJCB12. Again, 10 colonies for each mutant were selected, and *gyrA* was amplified and sequenced. Two mutants containing the desired mutations (Ser83 or Asp87 substitution) were randomly chosen; these were named L821 and L825, respectively. The absence of any other mutations within *gyrB*, *parC*, and *parE* in L825 was also confirmed by sequencing. Inactivation of *emrAB* was done by P22 transduction of a mutant allele with a chloramphenicol insertion within *emrAB* from strain EG16571 (46) and selection of transductants with agar containing 25 µg/ml of chloramphenicol followed by PCR verification of the insertion as described elsewhere (46).

### Susceptibility testing.

Antimicrobial susceptibility was determined using the agar doubling dilution method according to the recommendations of the British Society for Antimicrobial Chemotherapy (47). Biolog experiments used the Phenomicroarray system as per the manufacturer’s recommendations and as described previously (48). Average respiration values were compared for significance between strains at the 26- and 48-h time points and were considered significant when for at least two of the four concentrations of test compound, a significant difference compared to SL1344 by Student’s *t* test was observed.

### Growth kinetics.

Growth of each strain was measured automatically using a BMG FluoSTAR Optima (BMG Labtech, United Kingdom). Eight replicate 5-ml LB cultures of each strain were inoculated by the addition of 50 µl from an overnight culture, and 200 µl of each culture was then added to a 96-well microtiter tray. The absorbance of each well was measured every 10 min at 600 nm for 16 h, and antibiotics were injected automatically from stock solutions to a final concentration of one-half the corresponding MIC against SL1344 after 180 min of growth. Data were analyzed using Microsoft Excel, and average growth graphs were plotted. Anaerobic growth was measured by inoculating triplicate 5-ml portions of LB broth as described above for each strain in a universal tube; these were then incubated in an anaerobic jar for 24 h under anaerobic conditions before the final optical density (OD) achieved by each strain was measured in a spectrophotometer at 600 nm.

### Flow cytometry.

Overnight cultures (5 ml) of SL1344, L821, and L825 were grown in LB broth at 37°C, diluted to an OD (600 nm) of 0.2 in fresh broth, and then used to inoculate fresh 5-ml portions of LB broth containing the desired concentration of ampicillin. For heat shock and ciprofloxacin shock experiments, cultures were diluted as described above and then either incubated at 42°C for 2 h or in the presence of one-half the SL1344 MIC of ciprofloxacin for 2 h before again being diluted to 0.2 and inoculated as above. After 30 min and 4 h of ampicillin exposure, 1 ml of each culture was removed, harvested by centrifugation, and resuspended in 500 µl of Dulbecco’s phosphate-buffered saline (PBS). An ethanol-killed sample of bacteria was used as a control and made by harvesting 1 ml of cells and then resuspending them in 500 µl of 100% ethanol for 10 min before harvesting again and resuspending in 500 µl PBS. To prepare the cells for the flow cytometer, 50 µl of the PBS-suspended cells were added to 1 ml of FACSFlow buffer (BD, United Kingdom) in a tube. A 10-µl portion of working concentrations of PI (propidium iodide) and BOX (bis-oxonol) were added to this, and then the mixture was analyzed with an Aria II fluorescence-activated cell sorter (BD, United Kingdom). Cells were illuminated with a 488-nm laser, and data from 10,000 particles were collected. Experiments were replicated with three separate biological replicates per strain or condition. Forward scatter (FSC) and side scatter (SSC) data were collected along with PI fluorescence (red; collected through a long pass [LP] 565 mirror and band pass [BP] 610/20 filter) and BOX fluorescence (green; collected through an LP 502 mirror and BP 530/30 filter). PI stains the DNA of bacteria that have lost membrane integrity, while BOX stains bacteria with a collapsed membrane potential. Data were analyzed with FACSDiva (BD); postcollection compensation was used to eliminate spectral overlap and was calibrated using singly stained ethanol-killed cells. Data were plotted using bi-exponential scaling. Particles smaller than bacteria were eliminated by adjusting the threshold values on FSC and SSC channels.

### Supercoiling assays.

pBR322 was introduced into each of the strains by electroporation to identify differences in supercoiling activity between strains (4). Strains carrying pBR322 were grown to mid-logarithmic phase (OD 0.5 to 0.6 at 600 nm) in 20 ml of LB broth containing 100 µg/ml of ampicillin (to maintain the plasmid) aerobically at 37°C with shaking at 150 rpm, 5 ml of culture was harvested, and plasmid DNA was purified with the Qiagen plasmid mini-spin kit according to the manufacturer’s instructions. Plasmid DNA (1 µg of DNA in a 20-µl final volume) was separated by gel electrophoresis on 1% agarose gels containing 25 mg/liter of chloroquine for 16 h at 50 V before being washed for 4 h in distilled water and run in a second dimension for a further 16 h. After electrophoresis, gels were washed with deionized water for 3 h to remove chloroquine before being stained with ethidium bromide (2.5 µg/ml in distilled water) for 1 h, briefly washed again with water, and visualized on an ultraviolet transilluminator. Images were captured and analyzed using GeneTools software (Syngene, Cambridge, United Kingdom). The median distances that the pBR322 topoisomers migrated were calculated based on densitometry plots.

### Fitness comparisons.

To determine the *in vitro* cost of the topoisomerase mutations present within L821 and L825, an adaptation of the method described by Komp Lindgren et al. (40) was used. Overnight cultures of SL1344, L821, and L825 were grown in 5 ml of LB broth at 37°C; these cultures were then diluted in fresh LB broth to an OD (600 nm) of 0.1. Fresh, prewarmed 100-ml aliquots of LB broth were then inoculated with an approximately 1:1 ratio of ~5 × 10^3^ CFU/ml of SL1344 and either L821 or L825 and incubated at 37°C. Four independent replicate cultures (from separate, initial cultures of SL1344, L821, and L825) were incubated and after 24 h used to inoculate a new batch of broth to initiate a second growth cycle under the same conditions; this was then done a third time. Aliquots of each broth were taken at 0 h and every 24 h subsequently, serially diluted, and plated onto LB agar and LB agar containing 25 µg/ml of nalidixic acid. Relative fitness was calculated from the selection coefficient (*S*) for each mutant, derived from the slope of the following: [(ratio of mutant to parent)/(number of generations/number of cycles)] × ln 2. The relative fitness of each mutant was given as 1 + *S*.

### RNA preparation and transcriptional analyses.

RNA was prepared from strains after inoculation of 24 ml of defined MOPS (morpholinepropanesulfonic acid) minimal medium (Teknova, United States) with 1 ml of overnight culture grown in Luria-Bertani broth as in our previous studies (49). Cultures were incubated at 37°C with shaking at 250 rpm. All cultures were grown in sterile 500-ml Erlenmeyer flasks. When the cultures had reached an OD at 600 nm (OD_600_) of ~0.7 (±0.02), 4 ml of culture was added to 1 ml of 5% phenol, 95% ethanol in a 50-ml Falcon tube and incubated on ice for 1 h. Cells were then harvested, and RNA was prepared using the Promega simian virus 40 (SV40) total RNA preparation kit according to the manufacturer’s instructions. The quantity and quality of RNA were determined using a Bioanalyzer (Agilent). Transcriptomic experiments used the pan-*Salmonella* generation IV microarray at the Sanger Genome Campus (Hinxton, United Kingdom). For microarray experiments, three cultures were grown for each strain, and two RNA preparations were made from each culture, giving a total of six test and six reference samples (three biological and two technical replicates of each). RNA (25 µg) was used to generate probes labeled with either Cy-3 or Cy-5 using Superscript III (Invitrogen, United Kingdom). For each microarray experiment, the parent (SL1344) RNA was pooled after quantification to provide a common reference. For each microarray experiment, six slides were hybridized with labeled probes, and three were hybridized with L821 labeled with Cy-3; the test strain was labeled with Cy-5 and three dye swaps. Hybridization and scanning were as described previously (49). Results were analyzed using Bioconductor as previously described (50, 51). Genes with B values (log odds value) of >0 and adjusted *P* values of <0.05 were taken as demonstrating significantly altered expression. Significantly differentially expressed gene lists were submitted to Visant (52) to identify gene products predicted to interact which were significantly altered in either or both mutants. Genes whose expression was potentially sensitive to structural changes in the chromosome were identified as those within clusters of at least ten genes where expression was altered in a consistent manner and were flanked by areas of AT-rich sequence within 500 bp of the cluster at both ends (identified qualitatively as areas with an AT content that was 2.5 standard deviations or greater than the chromosomal average using Artemis).

### Measurement of reactive oxygen species.

The indicator dye hydroxyphenyl fluorescein (HPF) was used to measure production of reactive oxygen species. Overnight cultures (10 ml) in LB broth were diluted to an OD_600_ of 0.3 in 10 ml of PBS before being incubated at 37°C with antibiotics at 50% of the MIC. Hourly, 1-ml aliquots were taken, and cells were harvested and washed twice in 1 ml of PBS. The fluorescence of drug-treated and untreated samples was measured at wavelengths of 492 nm (excitation) and 520 nm (emission) in a FluoSTAR Optima (BMG Labtech).

## SUPPLEMENTAL MATERIAL

Figure S1 Growth kinetics of SL1344, L821, and L825 in the absence of drug (A) or after addition of half the MIC of ampicillin (B), gentamicin (C), or triclosan (D). (E) Final ODs obtained for each strain after anaerobic inoculation. Download Figure S1, EPS file, 4.7 MB

Figure S2 (A) Average survival of populations of SL1344 treated with 25 µg/ml of ampicillin after heat shock or ciprofloxacin exposure (1/2 the MIC). BO indicates fluorescence after staining with bis-oxonol; PI indicates that after staining with propidium iodide. (B) Increase in viability of SL1344, L821, and L825 upon ampicillin treatment after heat shock. Download Figure S2, EPS file, 0.7 MB

Figure S3 Accumulation of Hoechst 33342 by SL1344, L821, and L825. Dye was injected at the third cycle (4.5 min), and accumulation was measured over a 35-min period. Download Figure S3, EPS file, 2.4 MB

Figure S4 (A) Survival of SL1344 after ampicillin treatment after preexposure to heat shock or a sublethal concentration of ciprofloxacin as assessed by flow cytometry. (B) Percentages of populations of each strain maintaining a membrane potential after exposure to ampicillin with or without a prior heat shock. (C and D) Survival of SL1344 and L825 after ampicillin exposure at 37 and 42°C, respectively, as measured by colony counting. Asterisks indicate values significantly different from that for the untreated control. Download Figure S4, EPS file, 1 MB

Figure S5 Production of reactive oxygen species after exposure to ampicillin or ciprofloxacin by all strains. Bars represent the average ratio of fluorescence produced by the reactive oxygen-sensitive dye HPF from antibiotic exposed cultures to that of antibiotic-free controls. Values are from 2 h after exposure to half the MIC of each drug. Asterisks indicate values that are statistically significantly different from that for SL1344. Download Figure S5, EPS file, 0.8 MB

Table S1 Genes significantly up-regulated in L825 relative to SL1344. Changes are relative to expression in SL1344; “B” values refer to log odds ratios. Table S1, DOC file, 0.3 MB.

Table S2 Genes significantly down-regulated in L825 relative to SL1344. Fold changes indicated are relative to expression in SL1344, “B” values refer to log odds ratios. Table S2, DOC file, 0.4 MB.
