# Supplementary material for: Clinically Relevant Mutant DNA Gyrase Alters Supercoiling, Changes the Transcriptome, and Confers Multidrug Resistance
Source: mBio. 2013 Jul 23;4(4):e00273-13. doi: 10.1128/mBio.00273-13 (PMC3735185; doi:10.1128/mBio.00273-13)
Supplement: Table S1 — Genes significantly up-regulated in L825 relative to SL1344. Changes are relative to expression in SL1344; “B” values refer to log odds ratios. [file mbo004131568st1.doc]

**Supplementary Table 1. Genes significantly up-regulated in L825 relative to SL1344.**

| **Gene** | **Name** | **Annotation** | **Fold change^a^** | **B value^b^** |
| --- | --- | --- | --- | --- |
| Respiration/energy generation | | | | |
| SL4118 | *aceB* | malate synthase A | 5.83 | 6.83 |
| SL3300 | *arcB* | aerobic respiration control sensor protein | 1.73 | 7.81 |
| SL0723 | *cydB* | cytochrome d ubiquinol oxidase subunit II | 2.45 | 16.24 |
| SL3984 | *fdoG* | formate dehydrogenase-O, major subunit | 4.85 | 67.46 |
| SL2075 | *galF* | UTP-glucose-1-phosphate uridylyltransferase | 2.59 | 24.64 |
| SL1683 | *galU* | glucose-1-phosphate uridylyltransferase | 2.49 | 20.56 |
| SL1176 | *icdA* | isocitrate dehydrogenase | 2.28 | 9.85 |
| SL2299 | *lrhA* | NADH dehydrogenase operon transcriptional regulator | 2.48 | 27.03 |
| SL0714 | *sdhC* | succinate dehydrogenase cytochrome b-556 subunit | 2.95 | 17.74 |
| SL1820 | *zwf* | glucose 6-phosphate dehydrogenase | 1.55 | 6.59 |
|  |  |  |  |  |
| Regulation/Stress response | | |  |  |
| SL3615 | *cspA* | cold shock protein | 118.17 | 73.42 |
| SL3451 | *dam* | DNA adenine methylase | 2.82 | 6.50 |
| SL2799 | *emrA* | multidrug resistance protein A | 6.03 | 72.29 |
| SL2798 | *emrR* | putative transcriptional regulator | 6.57 | 47.43 |
| SL1578 | *hslJ* | heat shock protein | 4.42 | 3.97 |
| SL2809 | *recA* | RecA protein | 2.83 | 36.22 |
| SL2604 | *rpoE* | RNA polymerase sigma-E factor (sigma-24) | 2.05 | 0.11 |
| SL3292 | *rpoN* | RNA polymerase sigma-54 factor (sigma-N) | 1.94 | 1.45 |
| SL2903 | *rpoS* | RNA polymerase sigma subunit RpoS (sigma-38) | 2.71 | 14.47 |
| SL3383 | *rpsD* | 30S ribosomal subunit protein S4 | 2.92 | 28.31 |
| SL4104 | *rsD* | putative regulatory protein | 11.17 | 21.82 |
| SL2603 | *rseA* | sigma-E factor negative regulatory protein | 1.95 | 17.67 |
| SL1405 | *rstA* | putative two-component response regulator | 2.53 | 0.20 |
|  |  |  |  |  |
| Protein synthesis | |  |  |  |
| SL0462 | *rpmE2* | putative 50s ribosomal protein L31 (second copy) | 34.44 | 3.52 |
|  |  |  |  |  |
| Transport |  |  |  |  |
| SL4331 | *cycA* | D-serine/D-alanine/glycine transporter | 2.58 | 26.62 |
| SL4238 | *dcuB* | anaerobic C4-dicarboxylate transporter | 11.40 | 6.04 |
| SL3472 | *feoA* | putative ferrous iron transport protein | 3.62 | 8.08 |
| SL3473 | *feoB* | ferrous iron transport protein B | 2.49 | 7.69 |
| SL0505 | *sfbC* | ABC transporter integral membrane protein | 5.59 | 19.66 |
| SL2841 | *sitA* | Iron transport protein, periplasmic-binding protein | 9.77 | 9.20 |
| SL1826 | *znuC* | high-affinity zinc uptake system ATP-binding protein | 10.57 | 47.72 |
|  |  |  |  |  |
| Pathogenesis | |  |  |  |
| SL0538 | *fimC* | fimbrial chaperone protein | 3.43 | 1.73 |
| SL0537 | *fimI* | fimbrin-like protein FimI (pseudogene) | 4.56 | 13.67 |
| SL0542 | *fimZ* | probable transcriptional regulator (FimXZ protein) | 5.47 | 0.60 |
| SL2850 | *orgAa* | oxygen-regulated invasion protein | 3.83 | 31.73 |
| SL2674 | *sopE* | invasion-associated secreted protein. | 4.52 | 0.67 |
| SL2674 | *sopE* | invasion-associated secreted protein. | 3.78 | 0.54 |
|  |  |  |  |  |
| Metabolism/biosynthesis | | |  |  |
| SL0915 | *aroA* | 3-phosphoshikimate 1-carboxyvinyltransferase | 2.44 | 16.66 |
| SL0737 | *aroG* | phospho-2-dehydro-3-deoxyheptonate aldolase | 3.58 | 29.38 |
| SL1281 | *aroH* | 3-deoxy-D-arabinoheptulosonate 7-phosphate synthase | 16.68 | 49.43 |
| SL3454 | *aroK* | shikimate kinase I | 2.08 | 24.07 |
| SL0383 | *aroL* | shikimate kinase II | 3.30 | 14.57 |
| SL2403 | *cysM* | cysteine synthase B | 4.09 | 15.88 |
| SL0530 | *cysS* | cysteinyl-tRNA synthetase | 1.94 | 3.25 |
| SL0214 | *dapD* | 2,3,4,5-tetrahydropyridine-2-carboxylate N- succinyltransferase | 2.85 | 29.13 |
| SL1426 | *dmsC* | putative dimethyl sulphoxide reductase subunit | 2.76 | 0.21 |
| SL0416 | *dxs* | 1-deoxyxylulose-5-phosphate synthase | 2.06 | 4.42 |
| SL1131 | *fabD* | malonyl CoA-acyl carrier protein transacylase | 1.77 | 5.29 |
| SL3044 | *fba* | fructose 1,6-bisphosphate aldolase | 2.99 | 28.92 |
| SL3420 | *fkpA* | FKBP-type peptidyl-prolyl isomerase | 2.55 | 14.33 |
| SL2170 | *folE* | GTP cyclohydrolase I | 2.17 | 15.55 |
| SL3266 | *folP* | dihydropteroate synthase | 1.78 | 7.97 |
| SL2962 | *gcvA* | regulatory protein for glycine cleavage pathway | 3.07 | 43.28 |
| SL2453 | *gcvR* | glycine cleavage system transcriptional repressor | 2.01 | 28.91 |
| SL1234 | *gdhA* | NADP-specific glutamate dehydrogenase | 3.72 | 29.34 |
| SL3829 | *glmU* | UDP-N-acetylglucosamine pyrophosphorylase | 1.75 | 9.24 |
| SL3954 | *glnA* | glutamine synthetase | 3.27 | 23.97 |
| SL0668 | *glnS* | glutaminyl-tRNA synthetase | 1.65 | 4.32 |
| SL4036 | *glpF* | glycerol uptake facilitator protein | 5.74 | 24.54 |
| SL3873 | *gppA* | guanosine-5-triphosphate,3-diphosphate pyrophosphatase | 4.09 | 20.54 |
| SL1383 | *gst* | glutathione S-transferase | 1.50 | 0.58 |
| SL3891 | *hemX* | uroporphyrinogen III methylase | 1.67 | 12.54 |
| SL3890 | *hemY* | Porphyrin biosynthetic protein | 2.00 | 7.10 |
| SL0764 | *hutI* | Imidazolonepropionase | 2.19 | 1.08 |
| SL3865 | *ilvA* | threonine deaminase | 1.94 | 2.52 |
| SL3863 | *ilvE* | branched-chain amino-acid aminotransferase | 2.09 | 9.35 |
| SL3862 | *ilvM* | acetohydroxy acid synthase II, small subunit | 6.25 | 66.31 |
| SL2991 | *lysA* | diaminopimelate decarboxylase | 3.41 | 2.03 |
| SL4156 | *lysC* | lysine-sensitive aspartokinase III | 6.08 | 32.57 |
| SL4234 | *melR* | melibiose operon regulatory protein | 25.88 | 14.76 |
| SL3919 | *metE* | 5-methyltetrahydropteroyltriglutamate methyltransferase | 6.45 | 18.77 |
| SL4123 | *metH* | homocysteine-N5- methyltetrahydrofolate transmethylase | 13.01 | 61.85 |
| SL3467 | *pckA* | phosphoenolpyruvate carboxykinase | 1.74 | 1.27 |
| SL0910 | *pflB* | formate acetyltransferase 1 | 3.44 | 16.70 |
| SL4069 | *ppc* | phosphoenolpyruvate carboxylase | 2.13 | 18.46 |
| SL0446 | *ppiD* | peptidyl-prolyl cis-trans isomerase D | 1.97 | 4.36 |
| SL2616 | *pssA* | CDP-diacylglycerol-serine O- phosphatidyltransferase | 3.11 | 14.06 |
| SL2307 | *pta* | phosphate acetyltransferase | 1.69 | 0.09 |
| SL2932 | *pyrG* | CTP synthetase | 1.60 | 8.30 |
| SL2072 | *rfbA* | TDP-glucose pyrophosphorylase | 3.09 | 4.69 |
| SL2074 | *rfbB* | dTDP-glucose 4,6-dehydratase | 1.62 | 0.00 |
| SL3038 | *serA* | D-3-phosphoglycerate dehydrogenase | 5.71 | 38.73 |
| SL0900 | *serS* | seryl-tRNA synthetase | 2.02 | 1.16 |
| SL0222 | SL0222 | undecaprenyl pyrophosphate synthetase | 2.60 | 1.20 |
| SL2123 | *thiD* | phosphomethylpyrimidine kinase | 3.20 | 2.59 |
| SL1267 | *thrS* | threonyl-tRNA synthetase | 2.75 | 16.00 |
| SL4030 | *tpiA* | triosephosphate isomerase | 1.68 | 7.40 |
| SL1657 | *trpB* | tryptophan synthase beta chain | 3.66 | 15.38 |
| SL1655 | *trpD* | anthranilate synthase component II | 49.89 | 33.83 |
| SL1654 | *trpE* | anthranilate synthase component I | 406.38 | 3.31 |
| SL4438 | *trpS2* | probable tryptophanyl-tRNA synthetase | 57.14 | 20.02 |
| SL4075 | *udhA* | possible pyridine nucleotide-disulphide oxidoreductase | 2.42 | 5.45 |
| SL3879 | *wecB* | UDP-N-acetyl-D-glucosamine 2-epimerase | 1.76 | 5.83 |
| SL0172 | *yadF* | carbonic anhydrase | 2.48 | 34.94 |
|  |  |  |  |  |
| Miscellaneous | |  |  |  |
|  |  |  |  |  |
| SL2265 | *ais* | ais protein | 1.95 | 20.75 |
| SL1851 | *cheY* | chemotaxis protein CheY | 2.45 | 1.93 |
| SL2056 | *cld* | polysaccharide chain length regulator | 2.45 | 9.90 |
| SL0676 | *fldA* | flavodoxin 1 | 1.84 | 3.33 |
| SL0131 | *ftsQ* | cell division protein FtsQ | 1.95 | 3.23 |
| SL3802 | *gyrB* | DNA gyrase subunit B | 2.76 | 33.85 |
| SL4296 | *hflX* | HflX protein, putative GTP-binding protein | 1.73 | 3.06 |
| SL3120 | *hybD* | hydrogenase-2 component protein | 3.29 | 9.40 |
| SL4112 | *hydH* | two-component system sensor protein | 3.31 | 7.12 |
| SL0094 | *imp* | organic solvent tolerance protein precursor | 1.75 | 6.53 |
| SL3259 | *infB* | protein chain initiation factor 2 | 3.47 | 50.56 |
| SL1268 | *infC* | translation initiation factor IF-3 | 2.06 | 14.60 |
| SL1666 | *ispZ* | putative intracellular septation protein | 1.46 | 2.25 |
| SL2167 | *mglB* | D-galactose-binding periplasmic protein precursor | 70.27 | 32.40 |
| SL2504 | *nifU* | NifU-like protein | 2.59 | 3.88 |
| SL3260 | *nusA* | L factor | 3.30 | 36.51 |
| SL3155 | *parE* | topoisomerase IV subunit B | 3.62 | 32.92 |
| SL3255 | *pnp* | polynucleotide phosphorylase | 2.21 | 34.63 |
| SL1774 | *prc* | tail-specific protease precursor | 2.12 | 3.86 |
| SL3017 | *prfB* | peptide chain release factor 2 (RF-2) | 2.23 | 11.94 |
| SL3876 | *rho* | transcription termination factor | 2.41 | 18.54 |
| SL0605 | *rna* | ribonuclease I precursor | 4.63 | 46.86 |
| SL4301 | *rnr* | ribonuclease R (RNase R) | 1.70 | 4.33 |
| SL0679 | *seqA* | seqA protein | 2.64 | 14.98 |
| SL0503 | *sfbA* | Lipoprotein | 8.74 | 60.21 |
| SL2660 | *smpB* | SsrA (tmRNA) binding protein | 4.74 | 39.55 |
| SL3375 | *sun* | sun protein | 2.12 | 1.20 |
| SL0728 | *tolR* | tolR protein | 3.26 | 14.98 |
| SL4170 | *ubiC* | chorismate lyase | 3.85 | 8.71 |
| SL3903 | *xerC* | integrase/recombinase | 3.07 | 21.02 |
| SL0654 | *ybeJ* | ABC transporter periplasmic binding protein | 5.52 | 40.73 |
| SL0879 | *ybjX* | putative virK protein | 4.48 | 39.43 |
| SL0905 | *ycaD* | probable transport protein | 3.29 | 1.21 |
| SL1825 | *yebL* | high-affinity zinc uptake system periplasmic binding protein | 71.46 | 81.53 |
| SL2502 | *yfhE* | chaperone protein HscB | 1.98 | 0.22 |
| SL3136 | *yghB* | DedA-family integral membrane protein | 1.91 | 0.13 |
|  |  |  |  |  |
|  |  |  |  |  |
| Putative/hypothetical/conserved | | |  |  |
| SL1968 | *SL1968* | putative putative prophage protein | 2.13 | 9.55 |
| SL2652 | *corB* | putative membrane protein | 4.98 | 8.71 |
| SL3729 | *mgtC* | conserved hyopthetical protein | 8.27 | 6.92 |
| SL2451 | *nlpB* | putative lipoprotein | 1.69 | 1.19 |
| SL0796 | *rhlE* | putative ATP-dependent RNA helicase rhlE | 8.45 | 18.78 |
| SL0082 | *SL0082* | probable secreted protein | 3.32 | 0.27 |
| SL0547 | *SL0547* | putative membrane protein | 30.32 | 10.85 |
| SL0846 | *SL0846* | putative membrane protein | 2.57 | 11.98 |
| SL0883 | *SL0883* | conserved hypothetical protein | 2.34 | 10.38 |
| SL0933 | *SL0933* | putative exported protein | 1.57 | 2.97 |
| SL1008 | *SL1008* | conserved hypothetical protein | 2.77 | 4.16 |
| SL1084 | *SL1084* | conserved hypothetical protein | 1.66 | 0.72 |
| SL1669 | *SL1669* | conserved hypothetical protein | 2.28 | 4.01 |
| SL1408 | *SL1408* | putative exported protein | 2.96 | 6.37 |
| SL1198 | *SL1198* | conserved hypothetical protein | 22.50 | 3.58 |
| SL1749 | *SL1749* | conserved hypothetical protein | 4.02 | 12.49 |
| SL1768 | *SL1768* | putative exported protein | 4.15 | 12.55 |
| SL2190 | *SL2190* | putative membrane protein | 1.69 | 0.12 |
| SL2250 | *SL2250* | putative transcriptional regulator | 3.35 | 0.51 |
| SL2300 | *SL2300* | putative aminotransferase | 3.07 | 2.04 |
| SL2317 | *SL2317* | putative glutathione-S transferase | 1.99 | 4.93 |
| SL2358 | *SL2358* | putative 3-ketoacyl-CoA thiolase | 18.46 | 5.03 |
| SL2368 | *SL2368* | conserved hypothetical protein | 3.38 | 0.80 |
| SL2395 | *SL2395* | phosphoenolpyruvate-protein phosphotransferase | 2.29 | 15.00 |
| SL2505 | *SL2505* | putative L-cysteine desulfurase | 3.07 | 17.28 |
| SL2848 | *SL2848* | hypothetical protein found within S. typhi pathogenicity island 1 | 2.99 | 3.94 |
| SL3035 | *SL3035* | conserved hypothetical protein | 1.86 | 5.26 |
| SL3041 | *SL3041* | conserved hypothetical protein | 2.22 | 1.36 |
| SL3072 | *SL3072* | conserved hypothetical protein | 3.44 | 4.49 |
| SL3079 | *SL3079* | possible oxygen-independent coproporphyrinogen III oxidase | 2.86 | 2.03 |
| SL3141 | *SL3141* | conserved hypothetical protein | 1.86 | 6.09 |
| SL3156 | *SL3156* | conserved hypothetical protein | 1.90 | 9.15 |
| SL3158 | *SL3158* | conserved hypothetical protein | 1.77 | 7.53 |
| SL3159 | *SL3159* | conserved hypothetical protein | 1.69 | 5.68 |
| SL3199 | *SL3199* | putative membrane protein | 1.79 | 0.12 |
| SL3252 | *SL3252* | probable amino acid permease | 72.42 | 19.66 |
| SL3253 | *SL3253* | ATP-dependent RNA helicase | 6.47 | 28.04 |
| SL3254 | *SL3254* | conserved hypothetical protein | 4.89 | 21.59 |
| SL3261 | *SL3261* | conserved hypothetical protein | 2.76 | 36.93 |
| SL3288 | *SL3288* | conserved hypothetical protein | 1.48 | 4.08 |
| SL3289 | *SL3289* | possible exported protein | 1.55 | 17.15 |
| SL3290 | *SL3290* | conserved hypothetical protein | 2.27 | 31.86 |
| SL3933 | *SL3933* | flavin reductases | 2.08 | 0.47 |
| SL3911 | *SL3911* | detergent-resistant phospholipase A | 1.85 | 1.34 |
| SL3949 | *SL3949* | hypothetical protein | 2.89 | 27.99 |
| SL3478 | *SL3478* | conserved hypothetical protein | 2.00 | 0.43 |
| SL3428 | *SL3428* | conserved hypothetical protein | 1.89 | 13.84 |
| SL4370 | *SL4370* | hypothetical protein | 21.09 | 10.47 |
| SL4371 | *SL4371* | hypothetical protein | 29.34 | 23.43 |
| SL4373 | *SL4373* | putative membrane protein | 7.90 | 2.86 |
| SL4440 | *SL4440* | conserved hypothetical protein | 3.20 | 23.22 |
| SL4446 | *SL4446* | putative membrane protein | 6.28 | 1.46 |
| SL4481 | *SL4481* | ferric iron reductase protein | 7.03 | 9.58 |
| SL0173 | *yadG* | hypothetical ABC transporter ATP-binding protein | 2.16 | 0.56 |
| SL0406 | *yajD* | conserved hypothetical protein | 1.80 | 2.48 |
| SL0467 | *ybaJ* | conserved hypothetical protein | 4.27 | 22.29 |
| SL0899 | *ycaJ* | conserevd hypothetical protein | 3.10 | 31.60 |
| SL0934 | *ycbL* | conserved hypothetical protein | 1.99 | 1.37 |
| SL4022 | *ydeW* | putative regulatory protein | 4.90 | 2.07 |
| SL1824 | *yebA* | conserved hypothetical protein | 25.35 | 39.73 |
| SL2408 | *yfeF* | putative oxidoreductase | 5.66 | 51.24 |
| SL2524 | *yfhA* | putative transcriptional regulator | 3.52 | 20.52 |
| SL2503 | *yfhF* | conserved hypothetical protein | 2.17 | 8.33 |
| SL2506 | *yfhP* | conserved hypothetical protein | 12.44 | 54.22 |
| SL2960 | *ygdE* | conserved hypothetical protein | 3.02 | 3.14 |
| SL3416 | *yheL* | conserved hypothetical protein | 2.32 | 3.89 |
| SL3419 | *yheO* | conserved hypothetical protein | 1.96 | 4.71 |
| SL3432 | *yhfA* | conserved hypothetical protein | 1.96 | 8.55 |
| SL3600 | *yhjW* | putative membrane protein | 6.84 | 7.23 |
| SL3613 | *yiaF* | conserved hypothetical protein | 1.59 | 2.16 |
| SL3771 | *yidF* | conserved hypothetical protein | 2.78 | 0.29 |
| SL4068 | *yijP* | putative membrane protein | 1.99 | 2.23 |
| SL4027 | *yneB* | putative aldolase | 2.34 | 2.02 |
| SL2997 | *yqeF* | probable acetyl-CoA acetyltransferase | 11.67 | 1.49 |
| SL3369 | *yrdC* | conserved hypothetical protein | 2.17 | 0.48 |
| SL3461 | *yrfE* | putative NUDIX hydrolase | 3.47 | 15.16 |
| SL3921 | *ysgA* | putative hydrolase | 1.46 | 0.43 |

^a^Fold change relative to SL1344, ^b^log odds ratio
