# Supplementary material for: Clinically Relevant Mutant DNA Gyrase Alters Supercoiling, Changes the Transcriptome, and Confers Multidrug Resistance
Source: mBio. 2013 Jul 23;4(4):e00273-13. doi: 10.1128/mBio.00273-13 (PMC3735185; doi:10.1128/mBio.00273-13)
Supplement: Table S2 — Genes significantly down-regulated in L825 relative to SL1344. Fold changes indicated are relative to expression in SL1344, “B” values refer to log odds ratios. [file mbo004131568st2.doc]

**Supplementary Table 2. Genes significantly down-regulated in L825 relative to SL1344**

| **Gene** | **Name** | **Annotation** | **Fold change^a^** | **B value** |
| --- | --- | --- | --- | --- |
| respiration/energy generation | | |  |  |
| SL0152 | *aceE* | pyruvate dehydrogenase E1 component | 0.37 | 20.42 |
| SL0153 | *aceF* | dihydrolipoamide acetyltransferase component (E2) of pyruvate dehydrogenase | 0.23 | 39.24 |
| SL3834 | *atpA* | ATP synthase alpha subunit | 0.42 | 34.98 |
| SL3831 | *atpC* | ATP synthase epsilon subunit | 0.38 | 15.74 |
| SL3832 | *atpD* | ATP synthase beta subunit | 0.33 | 35.12 |
| SL3837 | *atpE* | ATP synthase subunit C | 0.56 | 11.18 |
| SL3836 | *atpF* | ATP synthase subunit B | 0.68 | 3.86 |
| SL3833 | *atpG* | ATP synthase gamma subunit | 0.27 | 35.93 |
| SL3839 | *atpI* | ATP synthase protein I. | 0.33 | 3.62 |
| SL4369 | *cybC* | soluble cytochrome b562 | 0.23 | 49.97 |
| SL0435 | *cyoC* | cytochrome o ubiquinol oxidase subunit III | 0.07 | 48.26 |
| SL0434 | *cyoD* | cytochrome o ubiquinol oxidase C subunit | 0.05 | 15.66 |
| SL0433 | *cyoE* | cytochrome o ubiquinol oxidase C subunit | 0.03 | 43.39 |
| SL2118 | *fbaB* | fructose-bisphosphate aldolase class I | 0.24 | 5.47 |
| SL2181 | *fruA* | PTS system, fructose-specific IIBC component | 0.10 | 7.69 |
| SL2183 | *fruB* | pts system, fructose-specific IIA/FPR component | 0.07 | 12.21 |
| SL2182 | *fruK* | 1-phosphofructokinase | 0.15 | 12.65 |
| SL3303 | *gltD* | glutamate synthase (NADPH) small chain | 0.38 | 19.71 |
| SL3331 | *mdh* | malate dehydrogenase | 0.32 | 17.35 |
| SL3650 | *mtlA* | mannitol-specific enzyme II of phosphotransferase system | 0.28 | 26.84 |
| SL3651 | *mtlD* | mannitol-1-phosphate dehydrogenase | 0.31 | 20.36 |
| SL0733 | *nadA* | quinolinate synthetase A protein | 0.17 | 2.56 |
| SL4507 | *nadR* | conserved hypothetical transcriptional regulator | 0.63 | 2.59 |
| SL2229 | *napA* | probable nitrate reductase | 0.04 | 44.64 |
| SL2226 | *napB* | cytochrome c-type protein NapB precursor | 0.05 | 23.92 |
| SL2225 | *napC* | cytochrome c-type protein NapC | 0.11 | 4.47 |
| SL2231 | *napF* | ferredoxin-type protein NapF | 0.06 | 28.87 |
| SL1691 | *narH* | respiratory nitrate reductase 1 beta chain | 0.19 | 6.16 |
| SL1689 | *narI* | respiratory nitrate reductase 1 gamma chain | 0.34 | 0.36 |
| SL1693 | *narK* | nitrite extrusion protein (nitrite facilitator) | 0.02 | 4.92 |
| SL2488 | *ndk* | nucleoside diphosphate kinase (ndk) | 0.13 | 23.85 |
| SL3441 | *nirB* | nitrite reductase large subunit | 0.01 | 41.25 |
| SL3443 | *nirC* | putative nitrite transporter | 0.01 | 37.82 |
| SL3442 | *nirD* | nitrite reductase (NAD(P)H) small subunit | 0.02 | 48.67 |
| SL4213 | *nrfA* | cytochrome c552 precursor | 0.09 | 8.15 |
| SL4215 | *nrfC* | cytochrome c-type biogenesis protein | 0.14 | 4.10 |
| SL2291 | *nuoH* | NADH dehydrogenase I chain H | 0.33 | 25.15 |
| SL2289 | *nuoJ* | NADH dehydrogenase I chain J | 0.33 | 19.77 |
| SL2288 | *nuoK* | NADH dehydrogenase I chain k | 0.24 | 9.37 |
| SL2287 | *nuoL* | NADH dehydrogenase I chain L | 0.27 | 37.27 |
| SL2286 | *nuoM* | NADH dehydrogenase I chain M | 0.37 | 10.79 |
| SL2285 | *nuoN* | NADH dehydrogenase I chain N | 0.28 | 19.33 |
| SL4011 | *pfkA* | 6-phosphofructokinase | 0.56 | 2.25 |
| SL3045 | *pgk* | phosphoglycerate kinase | 0.32 | 30.09 |
| SL1140 | *ptsG* | PTS system, glucose-specific IIBC component | 0.20 | 33.95 |
| SL2394 | *ptsH* | phosphocarrier protein HPr | 0.29 | 44.95 |
| SL3294 | *ptsN* | nitrogen regulatory IIA protein | 0.49 | 13.45 |
| SL0718 | *sucA* | 2-oxoglutarate dehydrogenase E1 component | 0.32 | 42.38 |
| SL0719 | *sucB* | dihydrolipoamide succinyltransferase component (E2) | 0.27 | 19.13 |
| SL0720 | *sucC* | succinyl-CoA synthetase beta chain | 0.25 | 39.16 |
| SL0721 | *sucD* | succinyl-CoA synthetase alpha chain | 0.56 | 8.88 |
|  |  |  |  |  |
| Regulation/Stress response | | |  |  |
| SL4008 | *cpxR* | two-component response regulatory protein | 0.58 | 20.03 |
| SL2620 | *clpB* | ClpB protein (heat shock protein f84.1) | 0.20 | 1.32 |
| SL0442 | *clpP* | ATP-dependent clp protease proteolytic subunit | 0.55 | 7.39 |
| SL4267 | *groEL* | GroEL protein | 0.17 | 52.61 |
| SL4266 | *groES* | GroES protein | 0.22 | 38.26 |
| SL2654 | *grpE* | heat shock protein GrpE (heat shock protein b25.3) (hsp24) | 0.29 | 12.97 |
| SL3775 | *hslS* | heat shock protein B | 0.29 | 0.31 |
| SL3776 | *hslT* | heat shock protein A | 0.14 | 14.00 |
| SL0896 | *lrp* | leucine-responsive regulatory protein | 0.71 | 0.11 |
| SL1169 | *phoP* | transcriptional regulatory protein PhoP, regulator of virulence determinants | 0.62 | 0.87 |
| SL4229 | *pmrA* | two-component response regulator | 0.33 | 18.51 |
| SL3185 | *rpoD* | RNA polymerase sigma-70 factor | 0.68 | 5.30 |
| SL3707 | *rpoZ* | DNA-directed RNA polymerase omega chain | 0.51 | 2.22 |
| SL3756 | *uhpA* | two-component system response regulator protein | 0.34 | 0.48 |
| SL3556 | *uspA* | universal stress protein A | 0.41 | 12.60 |
|  |  |  |  |  |
| Protein synthesis | |  |  |  |
| SL4089 | *rplA* | 50S ribosomal subunit protein L1 | 0.29 | 29.28 |
| SL3404 | *rplB* | 50S ribosomal subunit protein L2 | 0.59 | 1.81 |
| SL3392 | *rplF* | 50S ribosomal subunit protein L6 | 0.61 | 4.65 |
| SL4088 | *rplK* | 50S ribosomal subunit protein L11 | 0.32 | 21.28 |
| SL4091 | *rplL* | 50S ribosomal subunit protein L7/L12 | 0.21 | 33.56 |
| SL3388 | *rplO* | 50S ribosomal subunit protein L15 | 0.45 | 7.19 |
| SL3400 | *rplP* | 50S ribosomal subunit protein L16 | 0.37 | 25.92 |
| SL3381 | *rplQ* | 50S ribosomal subunit protein L17 | 0.32 | 7.22 |
| SL3391 | *rplR* | 50S ribosomal subunit protein L18 | 0.53 | 0.62 |
| SL1270 | *rplT* | 50S ribosomal subunit protein L20 | 0.43 | 11.43 |
| SL3402 | *rplV* | 50S ribosomal subunit protein L22 | 0.44 | 11.74 |
| SL3401 | *rpsC* | 30S ribosomal subunit protein S3 | 0.30 | 21.93 |
| SL3390 | *rpsE* | 30S ribosomal subunit protein S5 | 0.51 | 16.81 |
| SL4324 | *rpsF* | 30s ribosomal protein S6 | 0.34 | 16.53 |
| SL3316 | *rpsI* | 30S ribosomal subunit protein S9 | 0.49 | 5.05 |
| SL3408 | *rpsJ* | 30S ribosomal subunit protein S10 | 0.57 | 4.21 |
| SL3256 | *rpsO* | 30S ribosomal subunit protein S15 | 0.20 | 25.28 |
| SL3398 | *rpsQ* | 30S ribosomal subunit protein S17 | 0.15 | 33.87 |
| SL4326 | *rpsR* | 30s ribosomal subunit protein S18 | 0.32 | 20.78 |
|  |  |  |  |  |
| Transport |  |  |  |  |
| SL0469 | *acrA* | acriflavin resistance protein A precursor | 0.01 | 121.37 |
| SL0867 | *artP* | arginine transport ATP-binding protein ArtP | 0.42 | 11.52 |
| SL3596 | *dppA* | periplasmic dipeptide transport protein precursor | 0.56 | 18.01 |
| SL3595 | *dppB* | dipeptide transport system permease protein DppB | 0.42 | 2.31 |
| SL3594 | *dppC* | dipeptide transport system permease protein DppC | 0.30 | 9.12 |
| SL3593 | *dppD* | dipeptide transport ATP-binding protein DppD | 0.57 | 5.87 |
| SL3592 | *dppF* | dipeptide transport ATP-binding protein DppF | 0.49 | 14.52 |
| SL2252 | *glpT* | glycerol-3-phosphate transporter | 0.52 | 2.70 |
|  |  |  |  |  |
| Pathogenesis | |  |  |  |
| SL1184 | *pagC* | outer membrane invasion protein | 0.20 | 14.10 |
| SL0616 | *pagP* | antimicrobial peptide resistance and lipid A acylation protein | 0.50 | 6.28 |
| SL1161 | *sifA* | putative virulence determinant | 0.09 | 7.65 |
| SL1328 | *spiA* | putative outer membrane secretory protein | 0.12 | 4.14 |
| SL1341 | *ssaH* | putative pathogenicity island protein | 0.02 | 62.98 |
| SL1342 | *ssaI* | putative pathogenicity island protein | 0.02 | 67.12 |
| SL1343 | *ssaJ* | putative pathogenicity island lipoprotein | 0.04 | 88.59 |
| SL1346 | *ssaL* | putative secretion system protein | 0.03 | 8.98 |
| SL1347 | *ssaM* | putative pathogenicity island protein | 0.04 | 18.73 |
| SL1349 | *ssaN* | putative type III secretion ATP synthase | 0.09 | 34.72 |
| SL1350 | *ssaO* | putative type III secretion protein | 0.16 | 4.26 |
| SL1351 | *ssaP* | putative type III secretion protein | 0.12 | 16.05 |
| SL1354 | *ssaS* | putative type III secretion protein | 0.12 | 19.91 |
| SL1348 | *ssaV* | putative type III secretion protein | 0.05 | 18.50 |
| SL1333 | *sscA* | putative Type III secretion system chaperone protein | 0.06 | 45.68 |
| SL1337 | *sscB* | putative pathogenicity island protein | 0.07 | 17.96 |
| SL1331 | *sseA* | putative pathogenicity island protein | 0.02 | 35.52 |
| SL1332 | *sseB* | putative pathogenicity island effector effector protein | 0.06 | 50.99 |
| SL1334 | *sseC* | putative pathogenicity island effector protein | 0.08 | 58.43 |
| SL1335 | *sseD* | putative pathogenicity island effector protein | 0.03 | 62.65 |
| SL1336 | *sseE* | putative pathogenicity island effector protein | 0.04 | 56.35 |
| SL1338 | *sseF* | putative pathogenicity island effector protein | 0.11 | 4.52 |
| SL1326 | *ssrA* | putative two-component sensor kinase | 0.07 | 1.91 |
|  |  |  |  |  |
| Metabolism/biosynthesis | | |  |  |
| SL0481 | *adk* | adenylate kinase | 0.34 | 1.60 |
| SL4072 | *argB* | acetylglutamate kinase | 0.38 | 4.43 |
| SL3435 | *argD* | acetylornithine aminotransferase | 0.25 | 2.24 |
| SL3262 | *argG* | argininosuccinate synthetase | 0.46 | 3.15 |
| SL3332 | *argR* | arginine repressor | 0.31 | 7.83 |
| SL0770 | *bioA* | adenosylmethionine-8-amino-7-oxononanoate aminotransferase | 0.14 | 3.61 |
| SL0771 | *bioB* | biotin synthetase | 0.11 | 30.14 |
| SL0067 | *carA* | carbamoyl-phosphate synthase small chain | 0.19 | 1.46 |
| SL0068 | *carB* | carbamoyl-phosphate synthase large chain | 0.14 | 9.60 |
| SL4013 | *cdh* | CDP-diglyceride hydrolase | 0.44 | 31.01 |
| SL3306 | *codA* | cytosine deaminase | 0.21 | 6.65 |
| SL3444 | *cysG* | siroheme synthase | 0.14 | 21.63 |
| SL2098 | *dcd* | deoxycytidine triphosphate deaminase | 0.64 | 8.01 |
| SL1818 | *eda* | KHG/KDPG aldolase | 0.58 | 4.69 |
| SL3046 | *epd* | D-erythrose 4-phosphate dehydrogenase | 0.37 | 2.43 |
| SL3030 | *gcvH* | glycine cleavage system H protein | 0.20 | 14.44 |
| SL3029 | *gcvP* | glycine dehydrogenase (decarboxylating) | 0.23 | 26.06 |
| SL0313 | *gpt* | xanthine-guanine phosphoribosyltransferase | 0.45 | 4.84 |
| SL2471 | *guaA* | GMP synthase (glutamine-hydrolyzing) | 0.45 | 1.67 |
| SL2472 | *guaB* | inosine-5-monophosphate dehydrogenase | 0.26 | 18.18 |
| SL0141 | *guaC* | GMP reductases | 0.30 | 27.09 |
| SL0203 | *hemL* | glutamate-1-semialdehyde 2,1-aminomutase | 0.47 | 2.50 |
| SL2049 | *hisD* | histidinol dehydrogenase | 0.59 | 1.55 |
| SL2055 | *hisI* | phosphoribosyl-AMP cyclohydrolase | 0.51 | 3.65 |
| SL0112 | *leuB* | 3-isopropylmalate dehydrogenase | 0.41 | 10.12 |
| SL0154 | *lpdA* | dihydrolipoamide dehydrogenase | 0.55 | 2.34 |
| SL1397 | *manA* | mannose-6-phosphate isomerase | 0.54 | 2.97 |
| SL4038 | *menG* | menaquinone biosynthesis protein | 0.65 | 0.78 |
| SL1016 | *mgsA* | methylglyoxal synthase | 0.63 | 0.73 |
| SL0778 | *moaA* | molybdenum cofactor biosynthesis protein A | 0.16 | 19.79 |
| SL0779 | *moaB* | molybdenum cofactor biosynthesis protein B | 0.28 | 48.26 |
| SL0780 | *moaD* | molybdopterin converting factor, subunit 1 | 0.22 | 50.96 |
| SL0781 | *moaE* | molybdopterin converting factor, subunit 2 | 0.43 | 2.62 |
| SL1530 | *pcgL* | D-alanyl-D-alanine dipeptidase | 0.22 | 16.71 |
| SL3034 | *pepP* | proline aminopeptidase II | 0.61 | 4.54 |
| SL1164 | *pepT* | Aminotripeptidase | 0.40 | 5.19 |
| SL1159 | *potD* | spermidine/putrescine-binding periplasmic protein precursor | 0.46 | 7.23 |
| SL0854 | *potF* | putrescine-binding periplasmic protein precursor | 0.34 | 10.28 |
| SL4347 | *ppa* | inorganic pyrophosphatase | 0.55 | 7.56 |
| SL3439 | *ppiA* | peptidyl-prolyl cis-trans isomerase | 0.44 | 5.84 |
| SL0529 | *ppiB* | peptidyl-prolyl cis-trans isomerase B | 0.65 | 0.18 |
| SL4114 | *purD* | phosphoribosylglycineamide synthetase | 0.11 | 4.03 |
| SL1100 | *pyrC* | Dihydroorotase | 0.18 | 10.47 |
| SL4390 | *pyrI* | aspartate carbamoyltransferase regulatory subunit | 0.16 | 8.51 |
| SL2951 | *sdaB* | L-serine dehydratase 2 (L-serine deaminase 2) | 0.26 | 2.23 |
| SL0716 | *sdhA* | succinate dehydrogenase flavoprotein subunit | 0.24 | 40.15 |
| SL0002 | *thrA* | aspartokinase I/homoserine dehydrogenase I | 0.22 | 22.96 |
| SL0004 | *thrC* | threonine synthase | 0.47 | 4.42 |
| SL2461 | *upp* | uracil phosphoribosyltransferase | 0.12 | 53.79 |
|  |  |  |  |  |
| Miscellaneous | |  |  |  |
| SL2332 | *cvpA* | colicin V production protein (DedE protein) | 0.19 | 14.79 |
| SL2333 | *dedD* | DedD protein | 0.69 | 5.27 |
| SL0187 | *dksA* | dosage-dependent dnaK suppressor protein | 0.49 | 10.95 |
| SL0012 | *dnaK* | DnaK protein (heat shock protein 70) | 0.09 | 39.66 |
| SL1631 | *fabI* | enoyl-acyl-carrier-protein reductase (NADH) | 0.43 | 32.97 |
| SL1868 | *ftnA* | Ferritin | 0.33 | 20.50 |
| SL0133 | *ftsZ* | cell division protein FtsZ | 0.38 | 26.89 |
| SL2518 | *hmpA* | flavohemoprotein (haemoglobin-like protein) | 0.24 | 0.22 |
| SL4457 | *hsdR* | subunit R of type I restriction - modification system | 0.31 | 5.14 |
| SL4109 | *hupA* | histone like DNA-binding protein HU-alpha (NS2) (HU-2) | 0.46 | 12.86 |
| SL0445 | *hupB* | DNA-binding protein HU-beta | 0.33 | 24.14 |
| SL0898 | *lolA* | outer membrane lipoprotein carrier protein precursor | 0.57 | 4.14 |
| SL0444 | *lon* | Lon protease | 0.71 | 2.67 |
| SL2802 | *luxS* | autoinducer-2 production protein LuxS | 0.33 | 29.04 |
| SL0255 | *mltD* | membrane-bound lytic murein transglycosylase d precursor | 0.48 | 0.38 |
| SL3346 | *mreB* | rod shape-determining protein | 0.34 | 25.45 |
| SL4341 | *msrA* | peptide methionine sulfoxide reductase | 0.65 | 2.30 |
| SL1277 | *nlpC* | putative lipoprotein | 0.28 | 36.20 |
| SL0226 | *ompH* | outer membrane protein OmpH precursor | 0.66 | 1.02 |
| SL0808 | *ompX* | outer membrane protein x precursor | 0.58 | 7.20 |
| SL0731 | *pal* | peptidoglycan-associated lipoprotein precursor | 0.29 | 33.71 |
| SL4325 | *priB* | primosomal replication protein N | 0.31 | 21.52 |
| SL3559 | *prlC* | oligopeptidase A | 0.44 | 8.03 |
| SL2067 | *rfbH* | putative dehydratase RfbH | 0.60 | 1.33 |
| SL2060 | *rfbK* | Phosphomannomutase | 0.50 | 11.11 |
| SL2062 | *rfbN* | putative rhamnosyltransferase | 0.39 | 18.19 |
| SL0411 | *ribH* | 6,7-dimethyl-8-ribityllumazine synthase (riboflavin synthase beta chain) | 0.64 | 2.93 |
| SL0238 | *rof* | ROF protein | 0.20 | 16.03 |
| SL3264 | *secG* | protein-export membrane protein | 0.32 | 21.29 |
| SL3648 | *selA* | L-seryl-tRNA(Ser) selenium transferase | 0.44 | 0.52 |
| SL1377 | *slyB* | outer membrane lipoprotein SlyB precursor | 0.44 | 19.03 |
| SL4004 | *sodA* | manganese superoxide dismutase | 0.40 | 10.41 |
| SL3674 | *tdh* | threonine 3-dehydrogenase | 0.29 | 30.13 |
| SL0218 | *tsf* | elongation factor Ts | 0.55 | 7.40 |
|  |  |  |  |  |
| Putative/hypothetical/conserved | | |  |  |
|  |  |  |  |  |
| SL0869 | *SL0869* | conserved hypothetical protein | 0.73 | 0.26 |
| SL0457 | *amtB* | probable ammonium transporter | 0.19 | 2.55 |
| SL4514 | *creA* | conserved hypothetical protein | 0.64 | 1.67 |
| SL3696 | *dfp* | conserved hypothetical protein | 0.53 | 3.20 |
| SL3508 | *gntK* | putative gluconokinase | 0.18 | 3.98 |
| SL1027 | *pipB* | conserved hypothetical protein | 0.14 | 17.61 |
| SL0302 | *SL0302* | possible outer membrane adhesin | 0.41 | 1.02 |
| SL0692 | *SL0692* | conserved hypothetical protein | 0.54 | 4.35 |
| SL1018 | *SL1018* | conserved hypothetical protein | 0.39 | 13.44 |
| SL1019 | *SL1019* | conserved hypothetical protein | 0.40 | 6.86 |
| SL1025 | *SL1025* | putative membrane protein | 0.63 | 5.07 |
| SL1105 | *SL1105* | conserved hypothetical protein | 0.49 | 9.11 |
| SL1126 | *SL1126* | conserved hypothetical protein | 0.49 | 2.49 |
| SL1127 | *SL1127* | conserved hypothetical protein | 0.40 | 21.88 |
| SL1142 | *SL1142* | putative protein kinase C inhibitor | 0.54 | 16.13 |
| SL1147 | *SL1147* | conserved hypothetical protein | 0.65 | 1.12 |
| SL1664 | *SL1664* | conserved hypothetical protein | 0.48 | 11.92 |
| SL1649 | *SL1649* | hypothetical oxidoreductase | 0.50 | 9.60 |
| SL1628 | *SL1628* | hypothetical protein | 0.04 | 39.76 |
| SL1568 | *SL1568* | conserved hypothetical protein | 0.38 | 0.05 |
| SL1506 | *SL1506* | putative regulatory protein | 0.35 | 0.02 |
| SL1345 | *SL1345* | putative pathogenicity island protein | 0.04 | 41.02 |
| SL1344 | *SL1344* | putative pathogenicity island protein | 0.03 | 80.22 |
| SL1255 | *SL1255* | putative sodium:dicarboxylate symporter | 0.39 | 2.33 |
| SL1227 | *SL1227* | conserved hypothetical protein | 0.52 | 0.40 |
| SL1189 | *SL1189* | putative cytochrome | 0.24 | 15.74 |
| SL2035 | *SL2035* | conserved hypothetical protein | 0.64 | 3.54 |
| SL2114 | *SL2114* | conserved hypothetical protein | 0.09 | 6.54 |
| SL2303 | *SL2303* | putative phosphatase | 0.51 | 14.76 |
| SL2409 | *SL2409* | conserved hypothetical protein | 0.41 | 17.27 |
| SL2659 | *SL2659* | conserved hypothetical protein | 0.56 | 11.53 |
| SL2763 | *SL2763* | conserved hypothetical protein | 0.20 | 41.46 |
| SL2884 | *SL2884* | hypothetical protein | 0.24 | 8.87 |
| SL2885 | *SL2885* | hypothetical protein | 0.23 | 9.29 |
| SL2944 | *SL2944* | conserved hypothetical protein | 0.53 | 11.15 |
| SL3026 | *SL3026* | conserved hypothetical protein | 0.48 | 7.04 |
| SL3139 | *SL3139* | possible oxidoreductase | 0.45 | 1.14 |
| SL3169 | *SL3169* | conserved hypothetical protein | 0.38 | 17.71 |
| SL3202 | *SL3202* | putative membrane protein | 0.57 | 3.15 |
| SL3206 | *SL3206* | putative membrane protein | 0.42 | 24.07 |
| SL3240 | *SL3240* | possible lipoprotein | 0.57 | 8.70 |
| SL3246 | *SL3246* | conserved hypothetical protein | 0.57 | 0.52 |
| SL3299 | *SL3299* | conserved hypothetical protein | 0.59 | 5.09 |
| SL3319 | *SL3319* | conserved hypothetical protein | 0.67 | 0.00 |
| SL3653 | *SL3653* | hypothetical protein | 0.26 | 1.20 |
| SL3631 | *SL3631* | putative electron-transport protein | 0.33 | 0.53 |
| SL3465 | *SL3465* | heat shock protein | 0.46 | 5.98 |
| SL4196 | *SL4196* | putative type-I secretion protein | 0.23 | 2.63 |
| SL4204 | *SL4204* | putative xanthine/uracil permeases family protein | 0.24 | 2.29 |
| SL4253 | *SL4253* | conserved hypothetical protein | 0.50 | 9.27 |
| SL4377 | *SL4377* | hypothetical protein | 0.01 | 0.31 |
| SL4480 | *SL4480* | conserved hypothetical protein | 0.49 | 15.01 |
| SL1531 | *ugtL* | putative membrane protein | 0.04 | 52.97 |
| SL0239 | *yaeP* | conserved hypothetical protein | 0.27 | 8.50 |
| SL0378 | *yaiB* | conserved hypothetical protein | 0.43 | 0.78 |
| SL0386 | *yaiE* | conserved hypothetical protein | 0.57 | 2.39 |
| SL0401 | *yajC* | putative membrane protein | 0.54 | 14.91 |
| SL0478 | *ybaB* | conserved hypothetical protein | 0.45 | 2.51 |
| SL0602 | *ybdQ* | conserved hypothetical protein | 0.57 | 0.97 |
| SL0783 | *ybhL* | putative membrane protein | 0.55 | 12.39 |
| SL1834 | *yebC* | conserved hypothetical protein | 0.52 | 4.35 |
| SL1815 | *yebF* | putative exported protein | 0.45 | 7.55 |
| SL2204 | *yejL* | conserved hypothetical protein | 0.47 | 0.93 |
| SL3977 | *ygjN* | conserved hypothetical protein | 0.47 | 0.42 |
| SL3632 | *yiaJ* | putative transcriptional regulator | 0.33 | 4.36 |
| SL3859 | *yifE* | conserved hypothetical protein | 0.21 | 26.91 |
| SL4116 | *yjaB* | putative acetyltransferase | 0.32 | 5.00 |
| SL2230 | *yojF* | putative napAB assembly protein | 0.02 | 31.30 |
| SL1353 | *yscR* | putative type III secretion protein | 0.07 | 48.42 |
| SL4344 | *ytfP* | conserved hypothetical protein | 0.60 | 1.83 |

^a^Fold change relative to SL1344,  ^b^log odds ratio
